# Electromagnetic Interference from Left Ventricular Assist Device (LVAD) Inhibiting the Pacing Function of an Implantable Cardioverter-Defibrillator (ICD) Device

**DOI:** 10.1155/2018/6195045

**Published:** 2018-10-03

**Authors:** Sebhat Erqou, Robert L. Kormos, Norman C. Wang, Dennis M. McNamara, Raveen Bazaz

**Affiliations:** ^1^Department of Medicine, Alpert Medical School of Brown University, Providence, RI, USA; ^2^Department of Medicine, Providence VA Medical Center, Providence, RI, USA; ^3^Heart and Vascular Institute, University of Pittsburgh Medical Center, Pittsburgh, PA, USA

## Abstract

There is an increasing prevalence of patients with concomitant implantable cardioverter-defibrillators (ICDs) and left ventricular devices (LVADs). The potential for negative interactions between these continually evolving technologies is a valid concern. Previously reported interactions include inappropriate ICD therapy and interference with ICD telemetry function. Understanding the nature of such interactions and developing a comprehensive strategy to approach such situations are important. In this report, we describe a case of electromagnetic interference from LVAD inhibiting the pacing function of an ICD that was corrected by reprograming the device. We would encourage investigators to review patients with ICD and LVAD in their institutions in order to help assess the frequency and nature of these and other interactions.

## 1. Background

Compared to medical therapy, the left ventricular assist device (LVAD) has been shown to prolong survival in patients with advanced heart failure who are awaiting or are not candidates for transplant [1]. Since most patients with advanced heart failure already have implantable cardioverter-defibrillators (ICDs) by the time they get LVAD implants, there is an increasing number of patients with a concomitant ICD and LVAD. With the increasing prevalence of coexistence between ICDs and LVADs, the potential for negative interactions between these devices would presumably increase. To date, a number of cases of adverse electromagnetic interference (EMI) between LVAD and ICD have been reported [2–6]. However, only two case series involving a total of 54 patients with implanted ICD and LVAD have assessed these interactions systematically [7, 8]. The reported interactions include inappropriate ICD therapy and interference with ICD telemetry function involving St. Jude Medical, Boston Scientific, and Sorin devices [2–8]. Understanding the nature of such interactions and developing a comprehensive strategy to approach them are important. In this case report, we describe a patient with an LVAD-ICD interaction that resulted in ventricular oversensing and subsequent inhibition of pacing.

## 2. Case Report

**Figure 1:**
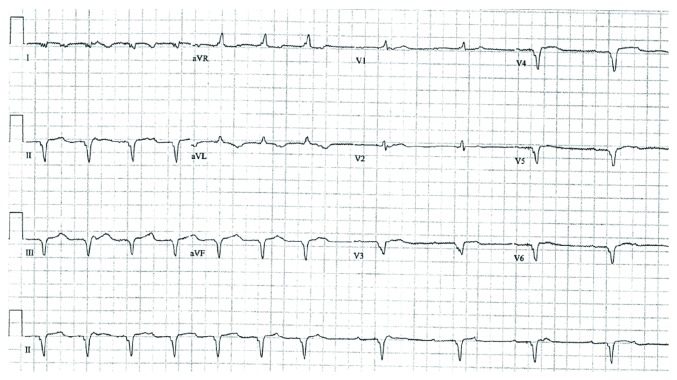
DDD pacing followed by VVI pacing showing an underlying rhythm of complete heart block.

A 60-year-old female with history of ovarian cancer, doxorubicin-induced dilated cardiomyopathy, and advanced heart failure had a Medtronic cardiac resynchronization-defibrillator (CRT-D) device placed in May 2010. The patient had implantation of HeartWare LVAD for destination therapy in August 2011 due to progression of heart failure and functional decline. The postsurgical course was complicated by ventricular tachycardia and multiple episodes of pump thrombosis. Interrogation of the CRT-D device in December 2011 (as well as subsequent device checks) revealed that the patient had evidence of complete heart block with no escape rhythm at ventricular backup rate of 40 (Figure 1 EKG). The CRT-D pulse generator was changed to a St. Jude Medical Assura model (3357-40C CRT-D) device in June 2014 once it reached its elective replacement indicator.

The patient was admitted to our hospital in November 2015 with concern for LVAD thrombosis. On this admission, the patient was treated with tissue-plasminogen activator (tPA) without improvement and was taken to the operating room for LVAD pump exchange on admission day 6. In the immediate postoperative period, it was noted that the patient's ICD was no longer consistently pacing. This prompted placement of epicardial pacing leads.

**Figure 2:**
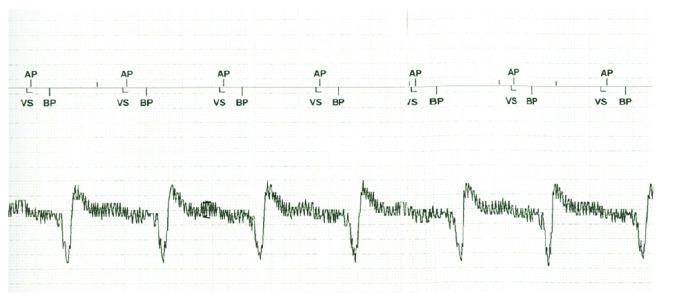
Pacing in asynchronous (DOO) mode with oversensing (marker annotations not affecting pacing mode) seen prior to the A paced marker.

Device interrogation revealed stable right ventricle (RV), left ventricle (LV), and shock lead impedances. The RV lead pace threshold was increased to 1.75 V at 0.5 ms (previously <1 V at 0.5 ms), while the LV lead pace threshold was stable at 0.75 at 0.5 ms. There was a continuous external noise with a mean amplitude of 1 mV detected by the ventricular sense amplifier of the RV lead. The intracardiac electrograms (IEGMs) showed an increase in a baseline noise signal compared to prior device-based recordings—the amplitude of which correlated with the increase of the LVAD pump rotation speed. The device was reprogrammed to nonsensing mode (DOO) allowing consistent pacer function of the ICD (Figure 2 IEGM). These setting changes meant that the tachytherapy function of the ICD was mandatorily disabled, as the device was set to a nonsensing mode.

The EMI oversensing issue was resolved by turning off the “low-frequency attenuation” (LFA) filter, which had resulted in amplification of the high-frequency VAD signal. This allowed the device to function in a sensing/tracking mode (DDD) without inhibition by noise from the LVAD with RV sensitivity threshold of 0.3 mV and permitted functional tachytherapy.

The LFA filter is a proprietary option in St. Jude Medical devices that suppresses low-frequency signals with the intent to mitigate T-wave oversensing. As with any “band-pass filter,” in addition to attenuating specific frequencies, they may also enhance or amplify other event frequencies, such as EMI, myopotential, R-waves, and far P-waves. The new VAD cannula position/orientation resulted in injection of the EMI signal more efficiently in the sensing antenna of the existing ICD lead. Additionally, the “sensibility” setting on the St. Jude device decays to baseline maximum sensitivity just prior to the A paced event—thus explaining why EMI was most consistently sensed in this window.

The patient underwent a successful defibrillation threshold (DFT) testing 10 days after her VAD exchange surgery. DFT testing showed that the ICD detected VF adequately with minimal dropout at a minimal sensitivity test setting of 1 mV. There was no further problem with the ICD, and the patient was discharged home one week later.

## 3. Discussion

To our knowledge, this is the first report where external noise from the LVAD device inhibited the pacer function of an ICD. This was caused by a combination of the new LVAD in-flow cannula position, as well as amplification of EMI by the LFA filter from the ICD. Previously reported interactions include inappropriate ICD therapy and interference with ICD telemetry function, as well as alteration RV sensing and pacing threshold [2–8]. Chhabra et al. [3] and Mozes et al. [6] have independently reported two cases of inappropriate tachytherapy delivery due to adverse EMI between HeartWare LVAD and ICD. Mozes et al. solved the problem by implanting a new pace-sense lead in the RV outflow tract “as far as possible” from the LVAD impeller and capping the pace-sense component of the old RV lead while continuing to use the existing atrial lead and defibrillation coils. A number of other case reports [2, 4, 5] as well as one case series of HeartMate II LVAD [8] have described loss of telemetry function of the ICD device after placement of LVAD, in some cases necessitating ICD replacement.

In the present case, we did observe an increase in RV lead pace threshold as was reported by Foo et al. [7]. The present case is unique, however, due to observation of significant adverse EMI causing external noise and leading to oversensing and resultant inhibition of pacing. This necessitated initial placement of epicardial wires in the operating room after the exchange of the LVAD pump. We were able to temporarily restore the pace function by reprogramming the device to nonsensed mode and decreasing the sensitivity of the RV lead to 1 mV, which unfortunately did not allow for the tachytherapy function to be turned on.

The HeartWare LVAD is a continuous flow system in which the impeller is suspended within the pump through magnetic and hydrodynamic forces. Electromagnetic forces power the rotational mechanism of the impeller. These electromagnetic forces could lead to adverse EMI with ICD as described above. While this inference may be nonconsequential in some cases, they may also lead to clinically significant problems such as inappropriate ICD shocks and loss of ICD-programmer telemetry, as well as inhibition of RV pacing as observed in our cases. Some of these cases have been corrected by using shielding techniques [4, 5], while others have necessitated ICD replacement. In the present case, we resolved the problem by changing device programming. Generally, it may be prudent to assess for these interactions intraoperatively to circumvent a subsequent ICD performance issue that may not be resolved by simple reprogramming.

There is paucity of systematic study into ICD-LVAD interactions. Data from the present and the previous case reports demonstrate that such interactions, albeit not very common, can be consequential. It is important to elucidate detailed mechanisms for ICD-LVAD interactions and describe the various types of interactions in sufficient detail. It will also be useful for investigators to review patients with ICD and LVAD in their institutions to assess the frequency and nature of such interactions.

## 4. Conclusion

There is accumulating evidence for potential adverse ICD-LVAD interactions. In this report, we described a case of EMI from LVAD inhibiting the pacing function of an ICD that was corrected by reprograming the device. We would encourage investigators to review patients with ICD and LVAD in their institutions to assess the frequency and nature of such interactions.
